# Cyclodiode vs micropulse transscleral laser treatment

**DOI:** 10.1038/s41433-024-02929-1

**Published:** 2024-01-30

**Authors:** Monica Kelada, Eduardo M. Normando, Francesca M. Cordeiro, Laura Crawley, Faisal Ahmed, Sally Ameen, Niten Vig, Philip Bloom

**Affiliations:** 1 Imperial College School of Medicine, Imperial College London, London, UK; 2 ICORG, Department of Surgery & Cancer, Imperial College London, London, UK; 3 Western Eye Hospital, Imperial College Healthcare NHS Trust, London, UK; 4 UCL Institute of Ophthalmology, London, UK; 5 The Hillingdon Hospitals NHS Foundation Trust, London, UK

**Keywords:** Surgery, Health care

## Abstract

**Background:**

Continuous-wave transscleral cyclophotocoagulation (CW-TSCP) is usually reserved for advanced/refractory glaucoma. Micropulse transscleral laser therapy (MPTLT) utilises short energy pulses separated by ‘*off*’-periods. MPTLT is postulated to have fewer complications, but its relative efficacy is not known. The National Institute for Health and Care Excellence (NICE) has deemed the evidence supporting MPTLT use of inadequate quality, limiting its use to research. This study aims to evaluate MPTLT efficacy and safety compared to CW-TSCP.

**Methods:**

This 24-month follow-up retrospective audit included 85 CW-TSCP and 173 MPTLT eyes at a London tertiary referral centre. Primary outcome was success rate at the last follow-up; defined as at least 20% intraocular pressure (IOP) reduction with the same/fewer medications, and IOP between 6 and 18 mmHg. Secondary outcomes were acetazolamide use and success rates per glaucoma type. Safety outcomes were reported as complication rates.

**Results:**

By 24-months, mean IOP reduced from 34.6[±1.4]mmHg to 19.0[ ± 3.0]mmHg post-CW-TSCP (*p* < 0.0001); and from 26.1[±0.8]mmHg to 19.1[±2.2]mmHg post-MPTLT (*p* < 0.0001). Average IOP decreased by 45.1% post-CW-TSCP, and 26.8% post-MPTLT. Both interventions reduced medication requirements (*p* ≤ 0.05). More CW-TSCP patients discontinued acetazolamide (*p* = 0.047). Overall success rate was 26.6% for CW-TSCP and 30.6% for MPTLT (*p* = 0.83). Only primary closed-angle glaucoma saw a significantly higher success rate following CW-TSCP (*p* = 0.014). CW-TSCP complication rate was significantly higher than MPTLT (*p* = 0.0048).

**Conclusion:**

Both treatments significantly reduced IOP and medication load. CW-TSCP had a greater absolute/proportionate IOP-lowering effect, but it carried a significantly greater risk of sight-threatening complications. Further prospective studies are required to evaluate MPTLT compared to CW-TSCP.

## Introduction

Glaucoma is one of the leading causes of irreversible blindness worldwide [1]. Elevated intraocular pressure (IOP) may be associated with chronic progressive optic nerve neuropathy characterised by damage to the optic nerve head (ONH), loss of retinal ganglion cells and visual field loss [1]. Raised IOP is the main known modifiable risk factor for glaucoma; all current glaucoma therapies aim to slow or halt disease progression, either by increasing aqueous humour (AH) outflow or by decreasing its production [2].

The ciliary body (CB) consists of an anterior portion, the pars plicata, and posterior portion, the pars plana [3]. The pars plicata epithelium is the principal source of AH [4, 5]. Destruction of the CB secretory epithelium, via transscleral cyclophotocoagulation (TSCP), has a well-established role in the treatment of uncontrolled glaucoma [6]. CW-TSCP transmits laser energy through the overlying conjunctiva and sclera, to target ciliary epithelium and stroma where it has a coagulative effect; the contact probe causes compression of the tissues, aiding transmission by minimising absorption as the energy passes through the ocular surface [7].

The original form of CW-TSCP delivers laser energy in a constant manner while the laser is ‘on’ (i.e., being applied), leading to the term continuous-wave transscleral cyclophotocoagulation (CW-TSCP) [8]. When directed at the pars plicata, the 810 nm laser energy causes epithelium ablation, resulting in homogenous blanching and shrinking of the ciliary processes, reducing AH production [9]. CW-TSCP (often referred to in the UK as ‘cyclodiode’) was initially reserved for refractory glaucoma or eyes with poor visual potential due to reports of reduced visual acuity following treatment and its perceived risk of sight-threatening complications [10]. Later publications propose, and largely support, its use earlier in the treatment process [11, 12].

There is some evidence that CW-TSCP, and other earlier forms of CW-TSCP such as neodymium:yttrium aluminium garnet (NdYAG)-TSCP, may also act as an outflow procedure [13, 14]. This might be explained by damage to the CB that renders it more permeable to aqueous outflow.

A more recent modality of transscleral laser energy delivery, micropulse transscleral laser therapy (MPTLT), administers laser energy directed at the pars plana in a series of repetitive, short pulses separated by rest (‘off’) periods [15]. It is theorised that the ‘off’ periods allow adjacent tissues to dissipate heat energy, preventing them reaching coagulative threshold [15]; this has therefore led to the perception that micropulse is a ‘non-thermal’ laser treatment. MPTLT, proposed to be as effective as CW-TSCP with fewer complications, was widely adopted in the UK from 2016 in earlier disease and in better sighted eyes than had been routinely treated by CW-TSCP [16].

It has been proposed that MPTLT may also act in part on aqueous outflow, based initially on an experimental study of autopsy eyes that used discrete spots of applied laser energy as opposed to the swept form of treatment employed in MPTLT [17]. The authors suggested that MPTLT may cause contraction of longitudinal ciliary muscle fibres with tightening of the scleral spur, hence the as yet unsubstantiated postulation that this may possibly prevent or reverse Schlemm’s canal collapse, leading to potential increase in trabecular outflow.

While MPTLT has shown promising results in many studies, the National Institute for Health and Care Excellence (NICE) has deemed the quality of evidence surrounding MPTLT efficacy as inadequate, although there were no added concerns regarding safety [18]. The decision by NICE was appealed but the original decision upheld [19].

Issues leading to the finding of inadequate quality of evidence include considerable inconsistency between studies regarding reported outcomes and their criteria for treatment success/failure [7, 16]. Previous studies are also often limited by their sample size and initially there was little consensus on optimal treatment parameters, such as laser energy settings used [7, 8].

Since the introduction of a revised MPTLT probe footplate design (MicroPulse P3® device, IRIDEX, Mountain view, California, USA) and the new manufacturer recommended treatment parameters, there has been greater consensus on how treatment should be applied to maximise efficacy [20].

Following the 2021 NICE interventional procedures guidance [IPG692], it was recommended that in the UK MPTLT is only performed for research purposes; which NICE defines as procedures in the context of formal research studies with approved use from a research ethic committee [18, 21]. Since that time, UK glaucoma units have necessarily reverted to CW-TSCP.

In truth, the quality of evidence supporting the use of CW-TSCP is not dissimilar to that for MPTLT, but ‘cyclodiode’ has been used for so long (many years before NICE existed) and is so ingrained in treatment paradigms, that its use has not been questioned. It is interesting to speculate whether the NICE review of MPTLT was triggered by conjecture about a novel mechanism of action for MPTLT, leading to the impression that MPTLT was a new interventional therapy rather than a relatively minor refinement of an existing treatment.

There is a clear need for further and improved research comparing MPTLT and CW-TSCP treatment outcomes. Here, we present a retrospective study with the aim of evaluating the efficacy and safety of MPTLT compared to CW-TSCP. Additionally, we investigate other factors that may affect glaucoma treatment outcomes.

## Methods

### Study design

This retrospective audit investigated treatment results in patients who underwent MPTLT or CW-TSCP, between February 2013 to October 2021, at the Western Eye Hospital London. Cases were identified from the departmental electronic medical record system (Medisoft Ophthalmology, Medisoft Ltd, Leeds, UK). Approval for audit was granted by the Imperial NHS Foundation Trust Ethics Committee. Informed consent was waived due to the retrospective nature of the study. All specific patient identifiers were removed. The tenets set forth in the Declaration of Helsinki were observed.

### Eligibility criteria

Patients with any type and stage of glaucoma, with at least three months follow-up, were included. Patients who had undergone any surgical glaucoma intervention within one year prior to laser treatment or had any other intra-ocular procedure within two months prior to laser treatment were excluded. Eye laterality was randomly selected using an Excel-based tool in cases where both eyes were operated on.

### Laser intervention

Laser procedures followed standard protocols used at the Western Eye Hospital [22]. Most patients received subtenons local anaesthetic blocks with 2% lidocaine. At clinician discretion, some patients received either peribulbar block, general anaesthetic, or a combination.

CW-TSCP was performed using the IRIS Medical Oculight SLx system (IRIDEX, Mountain View, California, USA). Initial standardised settings were 1500 ms duration and 1500 mW power, titrating power down to avoid ‘pops’. All four quadrants were treated with ten applications per quadrant, sparing a clock hour (30 degrees) at each of the 3- and 9-o’clock meridians to spare long ciliary nerves and vessels. Applications were placed with the front of the probe at the anterior aspect of the CB, as identified by transillumination [10].

For MPTLT procedures, the Iridex Cyclo G6 Glaucoma Laser machine (IRIDEX, Mountain view, California, USA) was used with an P3^TM^ handpiece employing standardised pre-set powers of 2500 mW/cm^2^ and duty cycle of 31.3% (on for 0.5 ms, off 1.1 ms) [23]. Energy was applied by sweeping the probe in a continuous “sliding” motion. Time applied per hemisphere was 90 seconds for eyes with IOP of over 30 mmHg and 80 seconds for eyes with IOP under 30 mmHg, with a sweep velocity of one hemisphere per 10 seconds [22].

All patients received a sub-conjunctival injection with betamethasone 0.5 ml of 4 mg/ml at the conclusion of surgery, followed by dexamethasone drops 0.1% 4x/day for at least 4 weeks, longer at the discretion of the operating surgeon [22].

### Data

Baseline parameters measured included: age at operation, patient-identified gender, reported ethnicity, co-morbidities, glaucoma type, and details of previous ocular surgeries.

The following variables were reviewed pre-operatively, at 1 day, 1 week, and 1, 3, 6, 12, 18, and 24 months after treatment: IOP measured by Goldmann applanation tonometry, number/type of glaucoma medications, complications, repeat procedures, and the need for subsequent alternative glaucoma surgery. We aimed to collect data from appointment records as close as possible to the desired follow-up time where available.

### Surgical success

The primary outcome was treatment success, classified as success at the last available follow-up. Follow-up was categorised into success or failure. Success was defined as at least 20% IOP reduction from the pre-operative measurement resulting in an absolute IOP between 6 and 18 mmHg, with the same or fewer glaucoma medications [16, 24, 25]. Failure was defined as an inability to reach success at two consecutive visits, an increased number of glaucoma medications, and a need for an additional or alternative glaucoma surgery.

Definition of success required both a proportional IOP reduction and quantitative value within a set range, to prevent undue favourable results for eyes with pre-operative IOP within a normal range.

Secondary outcomes of efficacy were the proportion of patients who discontinued oral acetazolamide (Diamox®) use, post-laser number of repeat/further treatments, and success rate per glaucoma type. Patients requiring further surgery were included up until the last available follow-up before the next intervention.

Safety outcomes were the incidence of individual complications and the overall complication rate following both procedures. Individual complications included hypotony, persistent hypotony, macular oedema, uveitis, hyphaema, phthisis bulbi, corneal epithelial defect, corneal oedema/Descemet’s folds. Hypotony was defined as an IOP less than or equal to 5 mmHg. Persistent hypotony was defined as hypotony over two consecutive follow-up visits lasting more than 90 days, or hypotony leading to choroidal detachment/effusion [26]. The overall complication rate was defined as the proportion of patients that experienced at least one complication during the follow-up period.

### Statistical analysis

All statistical analyses were performed using GraphPad Prism analysis software. A *p* value of ≤0.05 was considered statically significant. All graphs were produced using GraphPad Prism, all tables were produced using Microsoft Excel.

When comparing two independent proportions, a z-test was used. Chi-square testing was used for categorical values. A Shapiro-Wilk test was used to test for normality of continuous data. For non-parametric data, a Wilcoxon pairs signed-rank test (paired samples) or a Mann–Whitney U test (independent samples) was used. Student’s paired *t* test was used for parametric data.

The probability of success was analysed using a Kaplan-Meier graph with subsequent Mantel-Cox testing.

## Results

Table 1 summarises patient demographic information, glaucoma aetiologies and prior interventions. A total of 85 eyes underwent CW-TSCP and 173 eyes underwent MPTLT. The MPTLT treatment arm had significantly more patients with primary open angle glaucoma (POAG), whereas, the CW-TSCP treatment arm had significantly more patients with neovascular and aqueous misdirection glaucoma. The two groups did not differ significantly in terms of age or self-declared gender.

**Table 1:** Baseline characteristics of CW-TSCP and MPTLT patients.

	CW-TSCP	MPTLT	*P* value
Number of eyes	85	173	
laterality	44 left, 42 right	96 left, 77 right	
Gender			
Male [n (%)]	42 (49.4)	97 (56.1)	0.31 *
Female [n (%)]	43 (50.6)	76 (43.9)	
Age [mean (±sd)] (y)	66.6 ±16.2	67.7 ±15.8	0.72 **
Ethnicity			
White	26 (30.6)	50 (28.9)	0.62 *
Black	9 (10.6)	23 (13.3)	
Asian	15 (17.6)	22 (12.7)	
Other/mixed	23 (27.1)	43 (24.9)	
Not stated	12 (14.1)	35 (20.2)	
Aetiology/diagnosis [n (%)]			
Primary open angle			
Primary juvenile glaucoma	1 (1.2)	0	0.150 *#*
Primary open-angle glaucoma	28 (32.9)	93 (53.8)	0.0016 #
Normal pressure glaucoma	1 (1.2)	2 (1.2)	0.99 #
Primary closed angle			
Acute angle closure	1 (1.2)	1 (0.6)	0.60 #
Chronic angle closure	3 (3.5)	7 (4.1)	0.84 #
Secondary open angle			
Post-surgical	2 (2.4)	9 (5.2)	0.29 #
Post-traumatic	4 (4.7)	3 (1.7)	0.17 #
Inflammatory	2 (2.4)	6 (3.5)	0.62 #
ICE syndrome	0	1 (0.6)	0.48 #
Pseudoexfoliative	1 (1.2)	9 (5.2)	0.12 #
Secondary closed angle			
Neovascular glaucoma	23 (27.1)	10 (5.8)	<0.00001 #
Aqueous misdirection	4 (4.7)	0	0.004 #
Silicone oil	1 (1.2)	4 (2.3)	0.54 #
Phacomorphic	2 (2.4)	2 (1.2)	0.47 #
Post-keratoplasty	0	1 (0.6)	0.48 #
Ocular hypertension	2 (2.4)	8 (4.6)	0.37 #
Not recorded/Undetermined	10 (11.8)	17 (9.8)	0.63 #
Prior glaucoma interventions [n (%)]			
Trabeculectomy	2 (2.4)	9 (5.2)	0.29 #
Drainage Implant	8 (9.4)	11 (6.4)	0.38 #
Endoscopic Cyclophotocoagulation	2 (2.4)	7 (4.1)	0.48 #
iStent	1 (1.2)	6 (3.5)	0.29 #
TSCP	9 (10.6)	6 (3.5)	0.02 #
PreserFlo	0	4 (2.3)	0.16 #

Baseline characteristics of patients undergoing CW-TSCP (85 eyes) and MPTLT (173 eyes).

* Chi-Squared, ** Mann–Whitney U test, # Z-test.

*CW-TSCP* continuous wave transscleral cyclophotocoagulation, *MPTLT* micropulse transscleral laser therapy.

Type II diabetes mellitus (T2DM) was present in 22.4% and 20.8% of CW-TSCP patients and MPTLT patients, respectively. Similarly, 51.8% of CW-TSCP patients and 46.2% of MPTLT patients had systemic hypertension. 25.9% of CW-TSCP patients and 18.5% of MPTLT patients also had dyslipidaemia. The proportion of patients with these co-morbidities was not significantly different between both groups (*p* > 0.05, Z-test).

### Effect on IOP

**Fig. 1:**
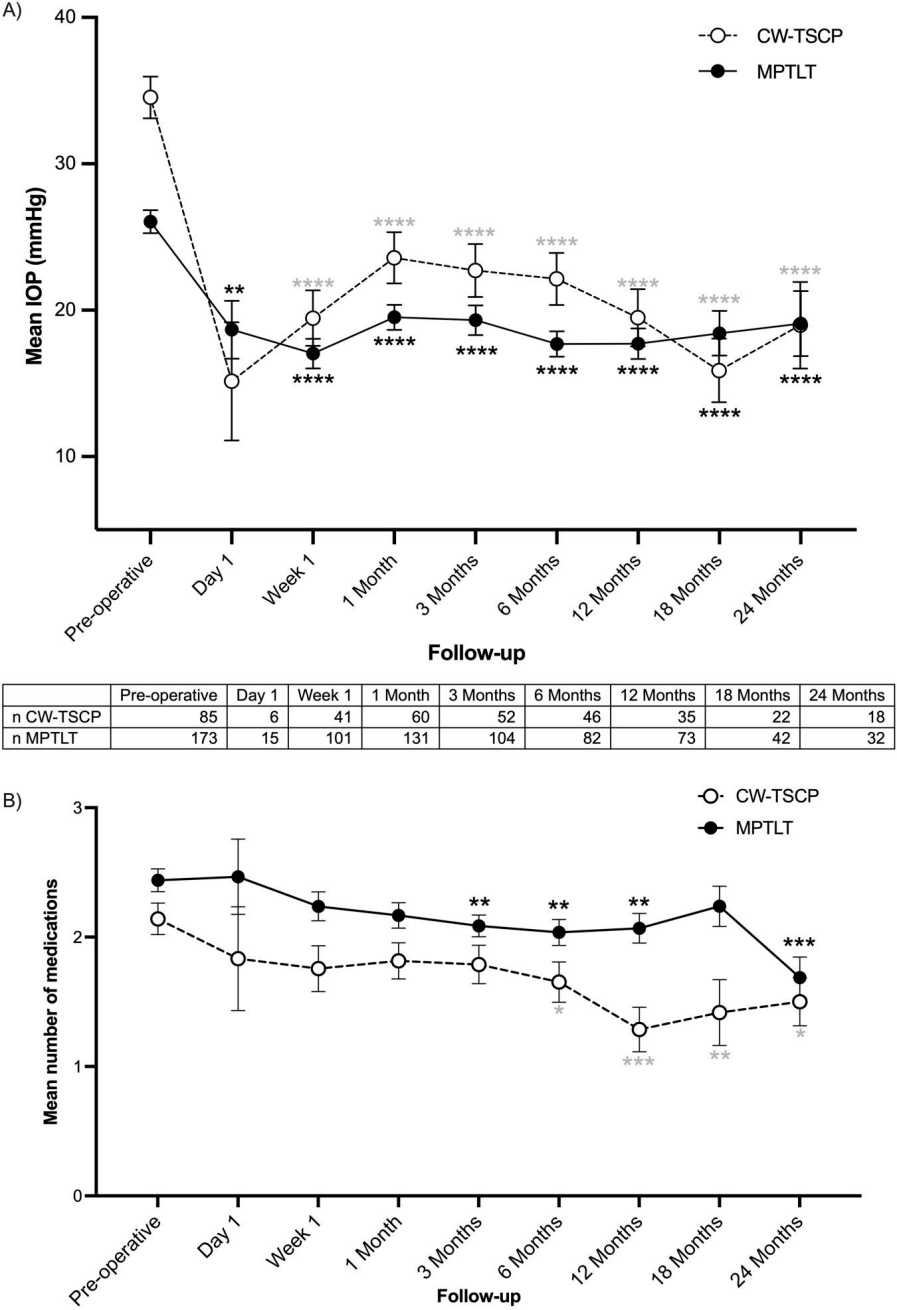
IOP and average number of glaucoma medications after CW-TSCP and MPTLT. **A** The effect of CW-TSCP (white circle) and MPTLT (black circle) on IOP. **B** The effect of CW-TSCP (white bar) and MPTLT (black bar) on the average number of glaucoma medications required. Vertical error bars represent SEM. *p ≤ 0.05, **p ≤ 0.01, ***p ≤ 0.001, ****p ≤ 0.0001—Wilcoxon matched pairs signed-rank test comparing pre-operative vs follow-up IOP after CW-TSCP (grey asterisks)/MPTLT (black asterisks). Paired *t*-test used for day-1, 3-months, 6-months MPTLT (parametric). Mann-Whitney U test comparing pre-operative and follow-up average number of medications after CW-TSCP (grey asterisks)/MPTLT (black asterisks). CW-TSCP continuous wave transscleral cyclophotocoagulation, MPTLT micropulse transscleral laser therapy, IOP intraocular pressure, n number of eyes at follow-up.

Following both CW-TSCP and MPTLT, IOP reduced significantly compared to baseline at all follow-up intervals up to 24 months (Fig. 1a).

After CW-TSCP, mean IOP reduced from 34.6[ ± 1.4] mmHg pre-op to 19.0[ ± 3.0] mmHg at 24 months (45.1% reduction); after MPTLT, mean IOP reduced from 26.1[ ± 0.8] mmHg pre-op to [19.1 ± 2.2] mmHg at 24 months (26.8% reduction)—see Fig. 1a.

### Effect on glaucoma medications

Figure 1b illustrates the average number of glaucoma medications throughout follow-up.

Both interventions significantly spared glaucoma treatments at 24 months (*p* ≤ 0.05); after CW-TSCP, medication use decreased from a mean of 2.1[ ± 1.1] to 1.5[ ± 0.2] agents, and after MPTLT, from 2.4[ ± 1.2] to 1.7[ ± 0.2] agents—see Fig. 1b.

In total, 57 eyes and 31 eyes undergoing MPTLT and CW-TSCP respectively, required acetazolamide pre-treatment. Significantly more of these patients were able to discontinue acetazolamide use after CW-TSCP (77.4%) compared to MPTLT (56.1%, *p* = 0.047).

### Overall treatment success

**Fig. 2:**
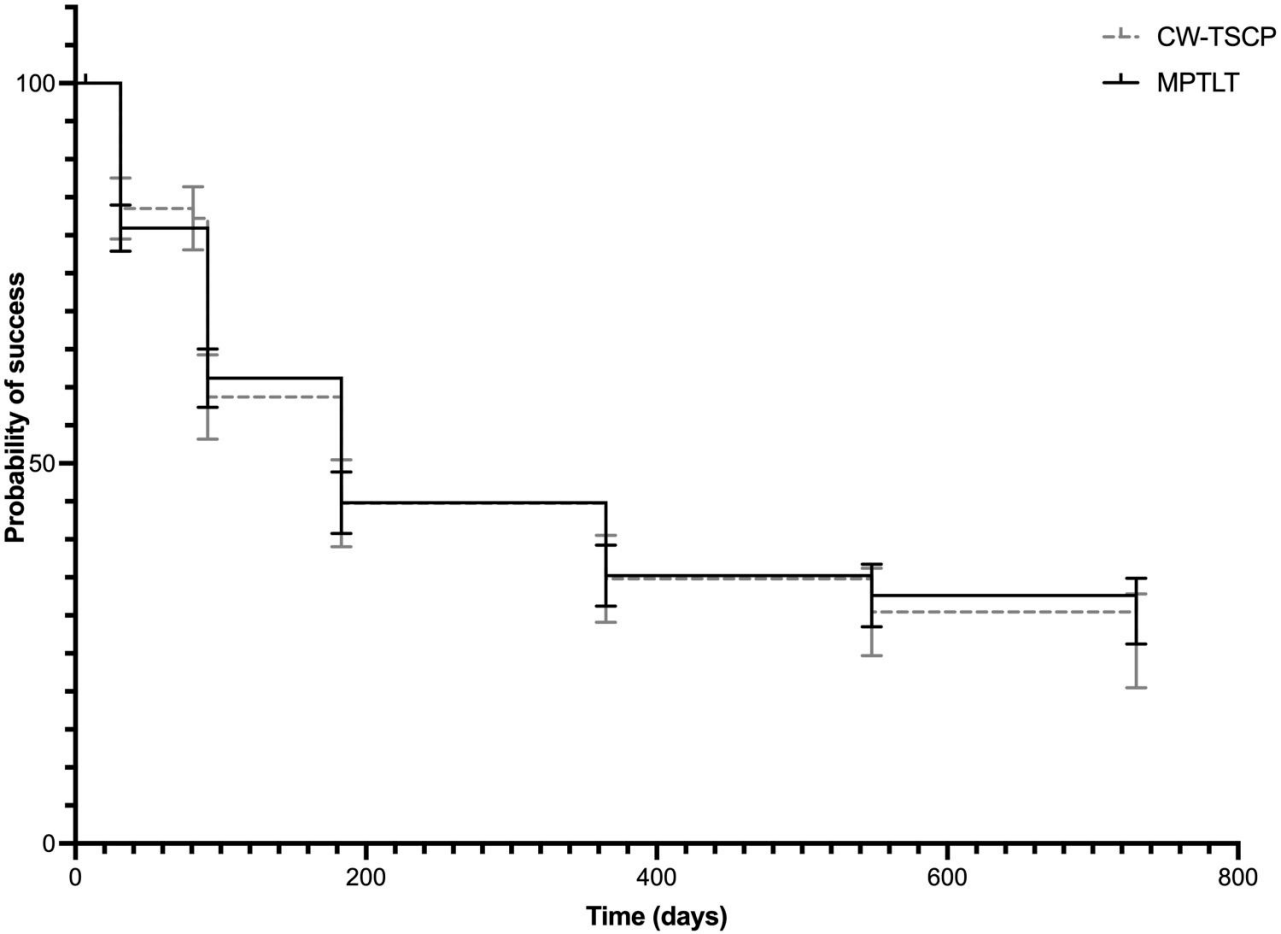
Kaplan-Meier analysis using cumulative probability of overall treatment success. Overall success: success at the last available follow-up. Success at follow-up: ≥ 20% IOP reduction AND IOP 6–18 mmHg, with the same or fewer glaucoma medications. Failure: an inability to reach either success criteria at 2 successive visits/an increased number of glaucoma medications/a need for an additional laser treatment/a need for alternative glaucoma surgery. 85 eyes underwent CW-TSCP, 173 eyes underwent MPTLT. Vertical bars represent SEM. Mantel-Cox test was used to test for significance between two curves (*p* = 0.94). CW-TSCP continuous wave transscleral cyclophotocoagulation, MPTLT micropulse transscleral laser therapy, IOP intraocular pressure.

Kaplan-Meier survival analysis showed comparable probability of overall success for both treatments by 24 months; for CW-TSCP 26.6[ ± 6.2]% and for MPTLT 30.6[ ± 4.3]%—see Fig. 2.

Significantly more CW-TSCP eyes received repeat laser treatment (21/85 eyes, 24.7%) compared to MPTLT (21/173 eyes, 12.1%), *p* = 0.01.

However, a significantly higher proportion of MPTLT (54/173, 31.2%) eyes required a further alternative glaucoma intervention post-treatment compared to CW-TSCP (9/85, 10.6%, *p* = 0.0003).

### Success rate per glaucoma type

Table 2 summarises the overall success rate for each glaucoma type. Patients with primary closed angle (PCA) had a significantly higher success rate following CW-TSCP compared to MPTLT.

**Table 2:** Summarising overall success for each glaucoma type.

Aetiology/diagnosis	CW-TSCP		MPTLT		P value
	Number of successfully treated eyes	Total number of eyes treated	Number of successfully treated eyes	Total number of eyes treated	
Primary open angle	17	30	44	95	0.32
Primary closed angle	4	4	2	8	0.014
Secondary open angle	2	9	10	28	0.45
Secondary closed angle	10	30	8	17	0.35
Ocular hypertension	0	2	4	8	0.2

Overall success: success at the last available follow-up.

Success at follow-up: ≥ 20% IOP reduction AND IOP 6–18 mmHg, with the same or fewer glaucoma medications. Failure: an inability to reach either success criteria at 2 successive visits/an increased number of glaucoma medications/a need for an additional laser treatment/a need for alternative glaucoma surgery.

85 eyes underwent CW-TSCP, 173 eyes underwent MPTLT.

*n* represents the number of eyes achieving success. The percentage was calculated as number of eyes reaching success/total number of eyes included in the study with corresponding glaucoma type.

Z-test significance comparing CW-TSCP and MPTLT success rate for each glaucoma type.

*CW-TSCP* continuous wave transscleral cyclophotocoagulation, *MPTLT* micropulse transscleral laser therapy.

### Safety outcomes

Table 3 illustrates the proportion of eyes experiencing each type of complication. There were no cases of phthisis bulbi and hyphaema after MPTLT. The most common complication for both treatments was hypotony, with incidence of 18.8% and 8.1% for CW-TSCP and MPTLT, respectively. Following CW-TSCP, one of the patients with persistent hypotony experienced choroidal detachment. One CW-TSCP eye required evisceration. CW-TSCP was associated with a significantly higher overall complication rate than MPTLT (33.3% v 17.6%, *p* = 0.0048).

**Table 3:** The percentage of eyes with complications following CW-TSCP and MPTLT.

	CW-TSCP	MPTLT	*P* value
Complication (%)			
Hypotony	18.8	8.09	0.011 *
Persistent hypotony	8.24	0.58	0.00084 ***
Macular oedema	3.53	2.9	0.78
Post-op uveitis	3.53	4.05	0.84
Hyphaema	1.17	0	0.15
Phthisis bulbi	3.53	0	0.013 *
Corneal epithelial defect	2.36	1.73	0.73
Corneal oedema/descemet’s folds	3.53	3.46	0.98

Hypotony = IOP ≤ 5 mmHg. Persistent hypotony = hypotony ≥ 2 consecutive visits lasting > 90 days or hypotony leading to choroidal detachment/effusion. CW-TSCP (white bar). MPTLT (black bar).

*CW-TSCP* continuous wave transscleral cyclophotocoagulation, *MPTLT* micropulse transscleral laser therapy.

*p ≤ 0.05, ***p ≤ 0.001 Z-test for significance in complication rate between procedures.

36.7% and 15.8% of POAG eyes had post-operative complications following CW-TSCP and MPTLT, respectively.

All PCA glaucoma eyes had complications after CW-TSCP, while only 25% had complications after MPTLT.

## Discussion

This study demonstrates that both CW-TSCP and MPTLT procedures significantly reduce IOP, with a greater absolute and proportional IOP reduction seen after CW-TSCP.

The day-1 rapid reduction seen after both procedures suggests that the immediate but transient effect may be secondary to active inflammation, which increases uveoscleral outflow and decreases AH production [27].

Although pre-operative IOP in the CW-TSCP cohort was significantly higher than MPTLT, average IOP by the end of follow-up was lower for CW-TSCP than for MPTLT. These results may indicate that CW-TSCP has a more efficacious IOP-lowering effect, but a further confounding factor may be that clinicians tend to titrate medical treatments to a target after surgical or laser treatment. Further studies with similar pre-operative IOP in both treatment arms would be helpful to further investigate this [16, 24].

The initial IOP was higher in the CW-TSCP group, which could indicate a selection bias if the treatment was reserved for more advanced cases.

At the end of follow-up, both treatments had a comparable probability of success (26.6[ ± 6.2]% for CW-TSCP, 30.6[ ± 4.13]% for MPTLT). Tan et al. defined success as at least 30% IOP reduction or an IOP between 6 and 21 mmHg and reported a 73% success rate for MPTLT [7]. Williams et al. reported MPTLT success rate of 66% and required at least 20% IOP reduction or IOP between 6 and 21 mmHg [28]. It is evident that heterogeneity in success criteria used between studies may contribute to the disparity in reported treatment success rates.

Most studies require either a minimum percentage IOP reduction or an absolute IOP (‘or’ criteria), while the present study requires both (‘and’ criteria) [7, 16, 28, 29]. Although ‘and’ criteria may place high expectations of success, this criterion may help reduce undue favourable results [30]. Patients starting and remaining within normal IOP ranges would be regarded as success by default, when using ‘or’ criteria [31].

Souissi et al. report a similar MPTLT success rate to our study, utilising similar success criteria [32]. However, the inclusion of only treatment naïve patients does not reflect common clinical practice [21, 33]. Additionally, while repeat treatments are common in clinical practice, the lack of reporting success rate per treatment course in many studies further limits comparison [34, 35]. Therefore, both unified success criteria and eligibility criteria are vital to allow valid comparisons across studies.

Comorbidities prevalent study cohorts, such as T2DM, may have affected treatment outcomes, given that they may be associated with a risk of elevated IOP [36–39]. A further study refinement would be to more closely match for such characteristics.

Another confounder could be ethnicity. Studies have reported higher inflammation rates amongst black and Asian patients [28, 40]. Williams et al. report 3.6 greater odds of prolonged inflammation in black patients, possibly associated with darker pigmentation leading to increased energy absorption [28]. In our study, 10.6% of CW-TSCP and 13.3% of MPTLT patients identified as ethnically black. Nevertheless, undue significance should not be placed on the effect of ethnicity given the underrepresentation of some ethnic groups in our cohort.

The precise IOP-lowering mechanism of action of MPTLT has not been fully elucidated. Suggested mechanisms for the effects of MPTLT include decreased AH production, increased uveoscleral outflow and increased trabecular outflow [41]. Some cadaveric studies have not shown significant CB histological changes, suggesting that the effects of MPTLT may be at least partly independent of its effects on AH production [42]. Barac et al. reported that successfully treated patients showed increased choroid thickness suggesting a role for increased uveoscleral outflow—however, their sample size (*n* = 22) was too small to determine statistical significance [43].

As mentioned previously, an experimental study by Johnstone et al. on monkey eyes reported shortening of the CB longitudinal muscles, which has been interpreted as showing that enlarged trabecular spaces may facilitate outflow [17]. Much has been made by the manufacturer of this theoretically different mode of therapeutic action, perhaps more than is justified on the limited evidence.

Many clinicians regard MPTLT as a refinement of CW-TCSP and were surprised when NICE saw fit to assess it as a ‘new treatment’, especially as it had already been in regular clinical use throughout the world for around 5 years. It is interesting to speculate whether the attempt to brand this treatment as different from CW-TSCP, was what prompted the NICE review, in which case it is a strategy that appears to have backfired.

Both CW-TSCP and MPTLT procedures significantly reduced need for glaucoma medications. Significantly more patients were able to discontinue acetazolamide use after CW-TSCP. Medication reduction is important to improve quality of life but also because medication adherence is often low and may account for substantial disease worsening [44].

It has been postulated that glaucoma sub-type may affect success rates for CW-TSCP [40, 45, 46]. If it is indeed the case that MPTLT achieves its effects by increasing outflow, it may be more effective in conditions such as POAG in which outflow pathway anatomy is relatively preserved. Conversely, this may be less effective in conditions in which there is obvious closure of the normal outflow pathways such as angle closure glaucoma or NVG [47]. Our study findings suggest that CW-TSCP is a more effective treatment than MPTLT for certain subtypes such as PCA glaucoma. However, given the small sample size for each glaucoma type outcomes should be interpreted with caution.

Previous studies report conflicting outcomes among NVG patients after MPTLT [7, 48]. Yelenskiy et al. reported higher success rates among POAG eyes than NVG eyes after MPTLT [8]. Although it has been suggested that NVG is prone to prostaglandin-mediated hypotony, no NVG patients in our cohort experienced hypotony after MPTLT [7]. It is well known that patients with NVG have ‘brittle’ IOP responses after any form of treatment, perhaps because outflow may be severely reduced due to secondary synechiae angle closure, with inflow also reduced but to a lesser extent [49]. This means that inflow treatments may disturb a delicate balance and lead to hypotony. This may explain the low success rate after CW-TSCP (21.7%) in eyes with NVG, given that 3 out of the 7 eyes with persistent hypotony suffered NVG [50].

In accordance with the results of previous studies, CW-TSCP demonstrated a significantly higher complication rate than MPTLT (33.3% vs 17.6%, respectively) [16, 20, 24]. Unlike CW-TSCP, none of the eyes in the MPTLT arm experienced phthisis bulbi or hyphaema. These results are in keeping with the concept that in CW-TSCP, prolonged laser absorption allows energy to spread to collateral tissues causing thermal damage and potential complications [51, 52].

In total, 36.7% and 15.8% of POAG eyes had post-operative complications following CW-TSCP and MPTLT, respectively. Previous studies, however, with shorter follow-up found no complications in POAG eyes after MPTLT [53].

Interestingly, all PCA eyes had complications after CW-TSCP compared to 25% seen post-MPTLT. Undue significance should not be placed on this finding given the small sample size of PCA eyes.

Limitations of our retrospective study include the disparity in the size of both arms reducing the statistical power of our study.

From 2013 to 16 only CW-TSCP was available; although both treatments were available from 2016 onwards, an increasing proportion of cases being treated with MPTLT after that time.

The COVID pandemic also adversely affected patient follow-up, limiting our ability to draw conclusions on mean IOP reduction. Virtual clinics limited the availability of IOP measurements [54]. Furthermore, during the pandemic MPTLT and CW-TSCP were commonly used as a temporizing procedure instead of more invasive surgeries [55]. This led to a change in treatment patterns with larger numbers of MPTLT procedures performed in situations when this treatment may not previously have been the first-line treatment choice. This may have affected its success rates and might have contributed to the observation that significantly more MPTLT patients required an alternative intervention.

## Conclusions

In conclusion, our study suggests that CW-TSCP is more efficacious in lowering IOP, with a greater absolute and proportionate IOP-lowering effect than MPTLT. However, this needs to be balanced against its increased risk of sight-threatening effects. Both treatments resulted in a significant and sustained IOP reduction, with similar overall success rates by our criteria. Both treatments facilitated a significant reduction in topical medication load, although a greater proportion of patients were able to discontinue acetazolamide use after CW-TSCP than after MPTLT.

We recognise the need for multi-centre prospective studies to more accurately evaluate the efficacy of MPTLT compared to CW-TSCP. We propose the adoption of standardised success criteria, such as those utilised in this study, to facilitate future comparisons.

## Summary

### What was known before

- Continuous-wave transscleral cyclophotocoagulation (CW-TSCP) is usually reserved for advanced/refractory glaucoma
- Micropulse transscleral laser therapy (MPTLT) utilises short energy pulses separated by ‘off’-periods. It is postulated that MPTLT is associated with fewer complications.
- NICE has deemed the evidence supporting MPTLT use of inadequate quality, limiting its use to research.

### What this study adds

- Both treatments significantly reduced IOP and medication load.
- CW-TSCP had a greater absolute/proportionate IOP-lowering effect.
- MPTLT had a significantly lower complication rate, while CW-TSCP carried significant risk of sight-threatening complications.
- This study adds to the evidence base for MPTLT, to try and resolve what NICE deems as ‘inadequate’ evidence.

## Data Availability

The data that support the findings of this study are not openly available due to reasons of sensitivity and are available from the corresponding author upon reasonable request. Data are located in controlled access data storage.
